# Operation for Hare-Lip

**Published:** 1856-10

**Authors:** Isham H. Ragan

**Affiliations:** Lithonia, Georgia


### ARTICLE II.

*Operation for Hare-Lip.* By Isham H. Ragan, M. D., of Lithonia, Georgia.

I was requested to operate for hare-lip on the child of Mr. J. C., of this county, aged about fifteen months. It had a single fissure on the left side, extending to the nostril; the left side of the superior maxillary bone being shorter and smaller than that of the opposite side. I first thought of adopting M. Nelaton's plan, as performed by Prof. Westmoreland, of Atlanta, and reported in the Nashville Journal of Medicine and Surgery, of February, 1855. Where this plan is practicable, it seems to have the advantage of most of others in many respects, especially in preventing the deformity (a notch or depression) so common after operations for hare-lip. But finding that there was not sufficient space between the angle of the fissure and the nose to make a continuous flap, I then determined to perform the operation according to the proposition of M. Malgaigne: that is, to fill the depression in the lip by two flaps made by two incisions, one on each side, commencing at the top of the fissure, and extending to within three lines of the border of the lip. These flaps are then reversed so as to bring the raw surfaces together, thus forming a projection below the border of the lip.

On the 9th of May last, assisted by some professional friends, I performed the operation according to the above plan. After making the incisions, I found that the textures were so closely adherent to the bone, that the opposite borders of the fissure could not be well adjusted without first slightly separating the lips from the bone, which I did with the point of the knife. Then, after bringing the flaps down, I brought the borders of the fissure together, and secured them by means of two silver pins, and secured the edges of the flaps with stitches. I then applied an adhesive strip between the two pins to extend from one cheek to the other, so that in crying or laughing, there would be less strain on the pins. I then gave directions to keep the child quiet, and should fever supervene, to use cold water freely to the wound.

On the 11th, I saw the child again, and found it doing very well; had had no fever of consequence. I took out the pins, and applied adhesive strips, letting the one applied the day of the operation remain. I saw it again in a few days, and found that it had adhered, leaving a very narrow cicatrix and a projection below the border of the lip of about two lines. Thinking the projection would remain too large, I drew a ligature around it so as to take off about one-third; and after the ligature had remained about twenty-four hours, I clipped it off with the scissors. The projection is now about one line in length, and seems to be gradually growing shorter. It will, perhaps, finally shrink away, so as not to be observable. It even now gives the lip a much more respectable appearance than a notch or depression would.

Should the projection still remain, after waiting sufficiently long for it to shrink away, I will then clip it off with the scissors.

